# Form and contour: breeding and genetics of organ shape from wild relatives to modern vegetable crops

**DOI:** 10.3389/fpls.2023.1257707

**Published:** 2023-09-28

**Authors:** Irwin L. Goldman, Yanbing Wang, Andrey Vega Alfaro, Scott Brainard, Madeline W. Oravec, Cecilia Elizabeth McGregor, Esther van der Knaap

**Affiliations:** ^1^ Department of Plant and Agroecosystem Sciences, University of Wisconsin-Madison, Madison, WI, United States; ^2^ Center for Applied Genetic Technologies, University of Georgia, Athens, GA, United States; ^3^ Department of Horticulture, University of Georgia, Athens, GA, United States; ^4^ Institute of Plant Breeding, Genetics and Genomics, University of Georgia, Athens, GA, United States

**Keywords:** market class, IQD/SUNs, fruit shape, root shape, TONNEAU1 Recruiting Motif (TRM) family, digital imaging, OVATE Family Proteins, universal shape regulators

## Abstract

Shape is a primary determinant of consumer preference for many horticultural crops and it is also associated with many aspects of marketing, harvest mechanics, and postharvest handling. Perceptions of quality and preference often map to specific shapes of fruits, tubers, leaves, flowers, roots, and other plant organs. As a result, humans have greatly expanded the palette of shapes available for horticultural crops, in many cases creating a series of market classes where particular shapes predominate. Crop wild relatives possess organs shaped by natural selection, while domesticated species possess organs shaped by human desires. Selection for visually-pleasing shapes in vegetable crops resulted from a number of opportunistic factors, including modification of supernumerary cambia, allelic variation at loci that control fundamental processes such as cell division, cell elongation, transposon-mediated variation, and partitioning of photosynthate. Genes that control cell division patterning may be universal shape regulators in horticultural crops, influencing the form of fruits, tubers, and grains in disparate species. Crop wild relatives are often considered less relevant for modern breeding efforts when it comes to characteristics such as shape, however this view may be unnecessarily limiting. Useful allelic variation in wild species may not have been examined or exploited with respect to shape modifications, and newly emergent information on key genes and proteins may provide additional opportunities to regulate the form and contour of vegetable crops.

## 1 From plant organs shaped by natural selection, humans have selected a diverse array of shapes in vegetable crops to satisfy consumer preferences

Shape is a primary determinant of consumer preference for many vegetable crops and it is associated with aspects of marketing, harvest mechanics, and postharvest handling (Peirce, 1991; Funk and Marshall, 2012). Perceptions of quality and preference often map to specific shapes for fruits, tubers, leaves, flowers, roots, and other plant organs, while certain crop shape abnormalities are signals of concern to vegetable consumers (Moser et al., 2011; Loebnitz and Grunert, 2018; Wiedmann et al., 2020). From crop wild relatives where fruits, tubers, and roots are the product of natural selection, humans have dramatically expanded the palette of shapes and sizes available for vegetable crops. In many cases, humans have created a series of market classes where particular shapes predominate (Luby et al., 2016). These shapes are funneled into market niches, where consumer preferences are specific and particular cultivars are recognized. To take but one example, the plethora of cultivars developed in recent centuries from non-pungent *Capsicum annuum* includes a wide range of market classes known as cherry, pimiento, sport, pepperoncini, Anaheim, banana, bell, cubanelle, and poblano types. These in turn represent a diverse array of shapes including round, cherry, blocky, bell, and berry, which are in widespread use in a variety of cuisines.

The human desire to use geometry to describe where we are and what things look like is primal (Ellenberg, 2021). Assessing the size, shape, and orientation of our surroundings is a constant draw on our conscious mind. And, when consciousness is altered by psychoactive substances, our minds conjure geometric shapes in startling colors and configurations (Bressloff et al., 2002). It appears that our desire to describe the shape and contour of our surroundings is a hard-wired part of our visual cortex (Bressloff et al., 2001). Food choices fit into these patterns, affirming the often-cited aphorism that “*we eat with our eyes*.” This adage has been experimentally verified, solidifying the relationship between visual stimuli and food shape (Delwiche, 2012). Experimental evidence from functional magnetic resonance imaging demonstrated that humans described curvilinear spaces as more beautiful than rectilinear spaces (Vartanian et al., 2013), and that the contemplation of curvilinear shapes exclusively activated the anterior cingulate cortex, a part of the brain associated with reward. Furthermore, activation of a neural network that underlies aesthetic evaluation of visual stimuli covaried with the perception of beauty. Munar et al. (2015) examined whether nonhuman great apes and humans exhibit visual preferences for curved contours using a forced choice experiment. They showed that humans’ preference for curved contours evolved from earlier primate species’ visual preferences, and suggest that it strengthened during human evolution as it became influenced by other cognitive processes. Although brain imaging studies on vegetable crop preferences have not yet been conducted, there is reason to suspect that food shape preferences are dictated to some degree by brain circuitry activated by visual stimuli.

**Figure 1:**
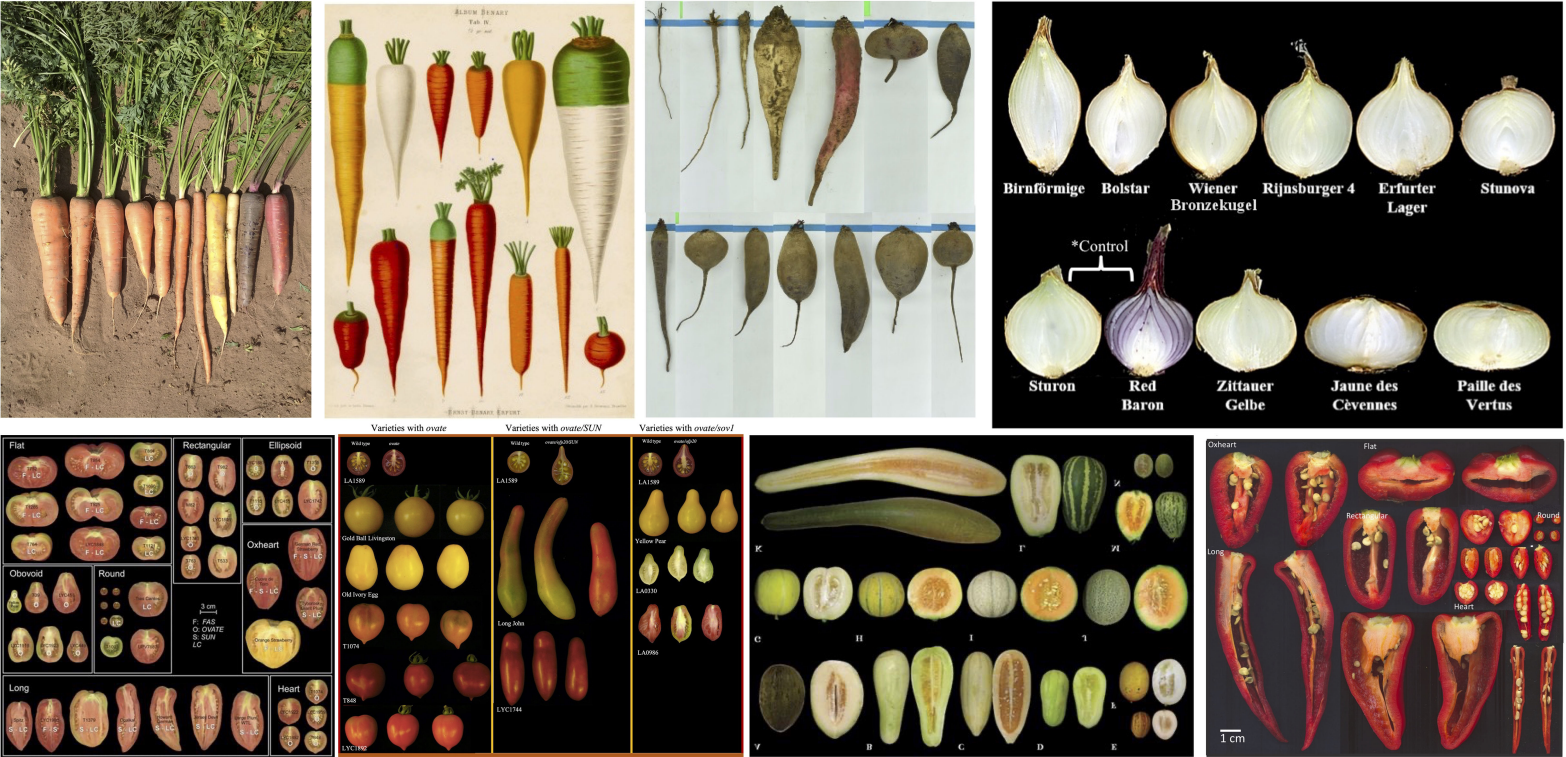
Clockwise from upper left: market classes in carrot (Goldman, I.), carrot (Source: Ernst Benary seed catalog, Erfurt, Germany, 1876), beet (Source: Oravec, M.), onion (Source: Boyhan, 1198), pepper (Source: Naegele et al., 2016), melon (Source: Monforte et al., 2014), tomato (Source: Monforte et al., 2014).

Many horticultural crops have been selected with a specific focus on the shape of the organ that is most sought by humans. As can be expected, crop domestication has resulted in traits that have disadvantages in a natural selection context (Dwivedi et al., 2023); shape and size among them. Here, we focus on vegetable crops where the shape of the organ that is consumed has been substantially modified from its wild progenitors, including our understanding of its regulation at the morphological, genetic, and molecular level. As the mutations arose during domestication and ensuing selection over thousands of years, alleles were repurposed to create a set of market classes. For horticultural crops, market class is perhaps best defined as a grouping of similar types that are available in the marketplace, such as russet potatoes, cut and peel carrots, or cherry or beefsteak tomatoes. Within each of these market classes, many cultivars may be available, each with different breeding programs focusing on specific production and aesthetic features (**Figure 1**).

A hallmark of artificial selection of crop wild relatives is the extreme modification of those plant organs that are of greatest interest to humans (Harlan, 1992). The profusion of modifications in leaves, roots, axillary buds, and floral organs in *Brassica oleracea* crops, resulting in cabbage, kohlrabi, Brussels sprouts, broccoli, cauliflower, and collards are an example of how particular plant organs have been targeted during domestication to create modern crops. Selection under domestication tends to exaggerate those traits of greatest interest to humans (Darwin, 1875). While the fruit-frugivore co-evolutionary relationship is well established (Lim et al., 2020), roots and tubers are less likely to be targets of herbivores. Nevertheless, roots and tubers in nature are likewise the product of natural selection and are therefore naturally selected for fitness traits such as water and nutrient uptake, carbohydrate storage, and physical support. Hominid evolution is characterized by an enhanced ability to digest starches, including seeds, roots, and tubers (Hardy and Brand-Miller, 2015; Fellows Yates et al., 2021), suggesting that these plant organs were among the earliest plant parts to be subjected to artificial selection. Artificial selection to modify these plant organs into vegetable crops is an example of how visual cues have driven crop evolution.

A parallel development by humans is selection for shape preferences in domesticated animals. Animal domestication followed many of the same pathways as plant domestication, satisfying human desires and heightening human preferences for particular traits. In a classic example, anatomical features of canines were shaped by artificial selection such that the muscle controlling raising of the inner eyebrow and widening the eyes is present in dogs but not wolves (Kaminski et al., 2019). Wolves possess only a small tendon for this purpose, and the movements that animate this muscle or tendon are of much greater intensity in dogs when they are in the presence of humans. Selection for these anatomical and behavioral modifications changed the contour of dog’s faces, making them more like human faces expressing sad emotions, and may therefore have promoted a nurturing response. Selection for body conformation in dairy cattle, including a suite of shape traits that were thought to predict productivity, has been practiced and studied for more than a century (Miglior et al., 2017). Charles Darwin famously used his understanding of the power of artificial selection on the shape of pigeons to develop his key ideas on evolution via natural selection (Desmond and Moore, 1991). Modifications to form and contour are among the most significant outcomes of artificial selection in both plants and animals.

### 1.1 Carrot and sweet potato

**Figure 2:**
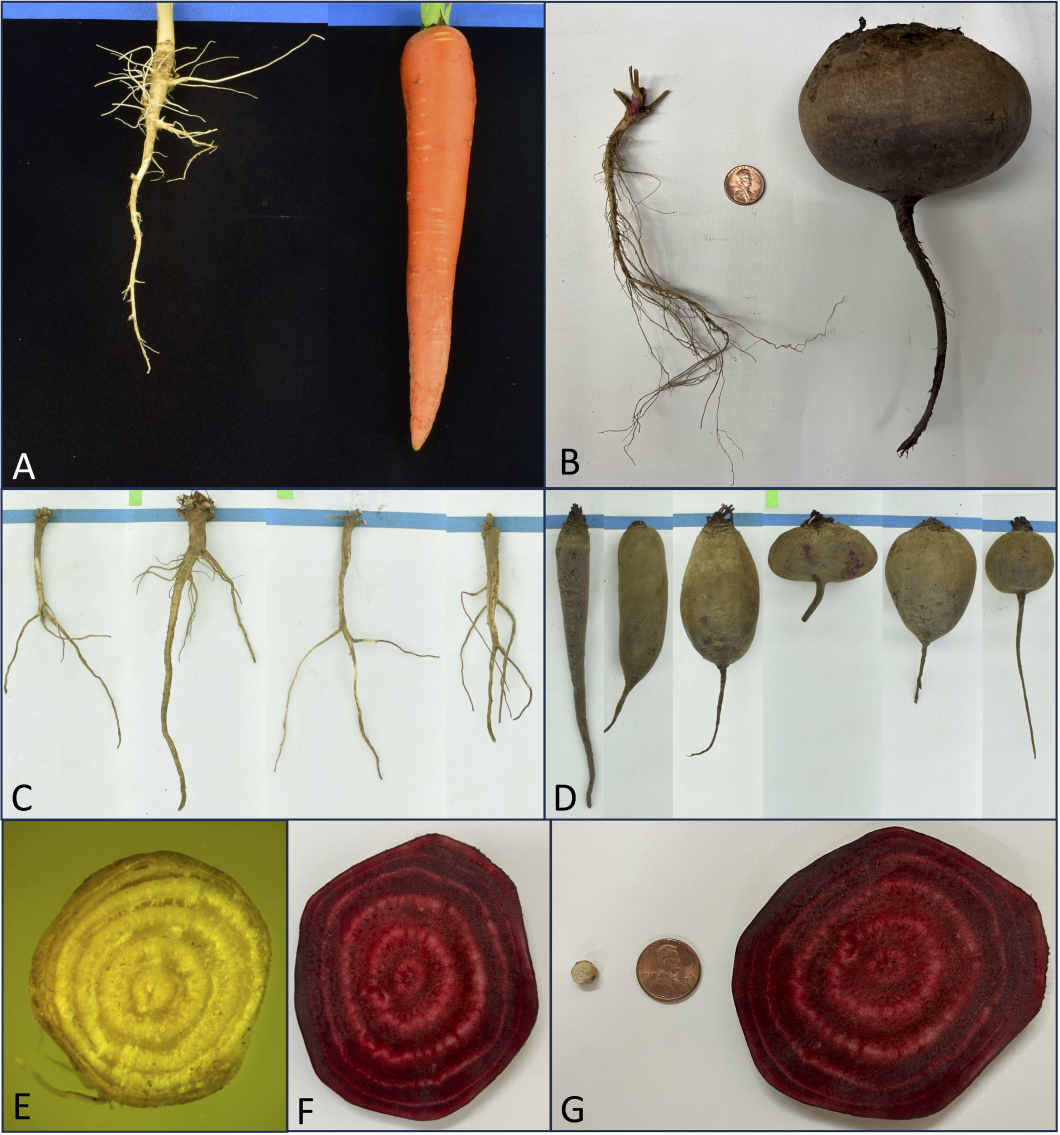
Roots of wild relative versus cultivated carrot and beet. **(A)** Typical roots from wild carrot *Daucus carota* var *carota*, also known as wild carrot or Queen Anne’s Lace (left) and a modern breeding line of a carrot cultivar (right) derived from a cross of between Imperator and Chantenay market classes. **(B)** Typical roots from cultivated beet, Beta vulgaris (right), and wild relative, Beta vulgaris subsp. maritima (left), and the range of roots shapes present in **(C)** wild and **(D)** modern table beet. Root cross sections showing supernumerary cambia apparent in **(E)** wild relative, Beta vulgaris subsp. maritima (PI 546509), and **(F)** cultivated beet and **(G)** the same cross sections of wild (left) and cultivated (right) beet with a penny for scale.

The genus *Daucus* in the *Apiaceae* family is native to central Asia. Wild carrot, *Daucus carota* var. *carota*, is a ubiquitous weed and the source of the domesticated vegetable. The wild carrot possesses only a very slightly swollen root that is often highly branched (**Figure 2**). In addition to modifications in life cycle, the most notable change in carrot domestication is the dramatic change in the size and shape of the root. Carrot is an example of a root crop where a large number of market classes- based primarily on shape- have been developed through breeding efforts (Mou, 2022). Examples of these include slim and elongated Imperator types for the cut and peel market, bulky Chantenay types for dicing, processing Danvers types for slicing and canning, cylindrical Nantes types for fresh eating, and Parisienne types for ball-shaped novelty carrots (Luby et al., 2016). There are between 10 to 15 recognized root shapes (types or market classes) in carrots today (Simon and Grezebelus, 2020). The different shapes often respond to different market needs and consumer preferences.

Sweet potato (*Ipomea batatas)* was domesticated in Mesoamerica from the wild progenitor *Ipomea trifida*, which is native to Central America and parts of South America. This wild relative possesses fibrous roots and, in certain cases, small, thickened roots (Komaki and Katayama, 1999), which were likely exploited during domestication. Root primordia may form adventitious roots, which have the potential to form storage roots under certain growing conditions. Adventitious roots may also become fibrous roots or pencil roots, the latter of which are only very slightly thickened and are not consumed. The storage root that is characteristic of the modern sweet potato forms when the cambium expands and the starch storage tissue proliferates (Eserman et al., 2018). This in turn dramatically expands the root and modifies the root shape. The modern sweet potato is an important starchy root vegetable in global diets, and the key step in its development approximately 4,000 years ago was selection for starch storage and corresponding swollen rootedness. The crop has not generally been selected into market classes defined by shape, although there are some generalized market classes recognized in certain markets. A few examples include garnet (elongated with orange flesh), Japanese (tapered with white flesh), Jewel (less elongated with orange flesh), and Beauregard (elongated with yellow flesh). In general, sweet potato market classes may be described by exterior and interior flesh colors, which range from white to yellow, orange, and purple, and culinary uses. Root shape is typically prolate spheroid, which is the shape of a football where the middle is large and the two ends are tapered. It is also possible to find cultivars that are more tubular in shape, with significantly less swelling in the middle region.

### 1.2 Beet and onion

The genus *Beta* in the family *Amaranthaceae* contains several important biennial *Beta vulgaris* crop species, including sugar beet, Swiss chard, mangel or fodder beet, and table beet. Wild *Beta* species (*Beta vulgaris* subsp. *maritima*) from the Mediterranean region possess roots with supernumerary cambia that expand during growth, but they are only slightly swollen and often fibrous (**Figure 2**). It appears that the first cultivated forms of this species were leaf vegetables, as described by the Romans, followed by swollen rooted forms that exhibited a biennial life cycle (Ford-Lloyd, 2005). The beet storage shapes include cylindrical, globe-shape, flat or Egyptian shape, round, and baby beets. Growth in the girth of beet and carrot roots is due to meristematic activity in the vascular cambium, producing xylem on the inner side and phloem on the outer side of the stem (Robert et al., 2011). Thus, the growth of this crop is due to secondary growth produced by secondary xylem and phloem, which represents a distinct growth form. Many vegetables are derived from extensive growth in leaf, stem, petiole, ovary, and fruit tissues, whereas storage roots are characterized by this unique form of secondary growth in the root and hypocotyl.

**Figure 3:**
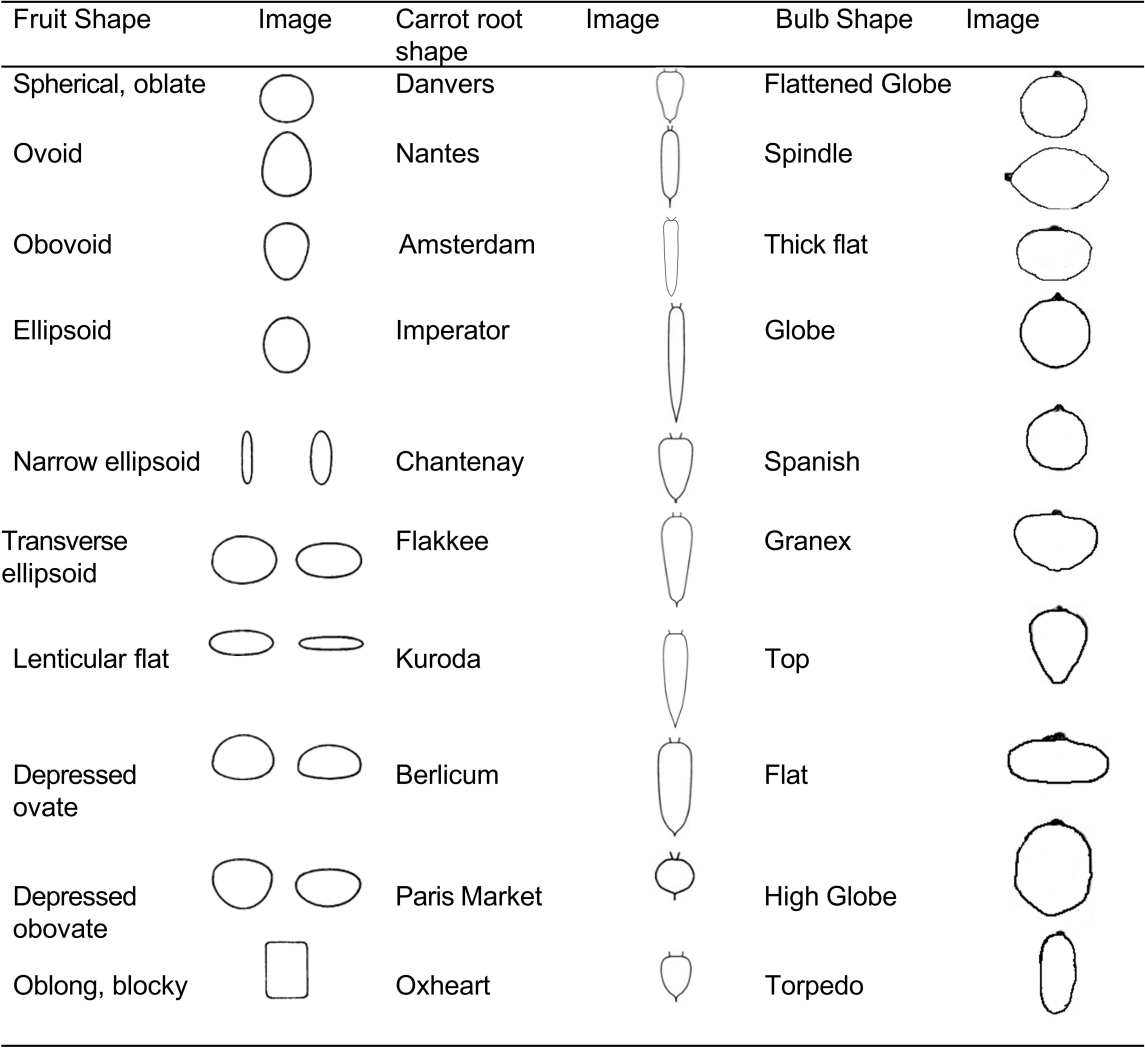
Common fruit, root, and bulb shapes in tomato, carrot, and onion. Sources: virtualherbarium.org, Virtual Herbarium, 2007; Simon, 2007; University of Georgia Onion Production Guide.

Bulb onion originated from progenitors in the Irano-Turanian region in Central Asia. Onion progenitor species are found in rocky sites with shallow soils, and typically have very long juvenile phases of 3-10 years prior to flowering (Brewster, 2008). The progenitor of bulb onion was rhizomatous, and evolution within the subgenus *Cepa*, to which bulb onion belongs, resulted in the formation of a vertical rhizome. This feature appears as a disc-like stem from which leaves originate. Onion bulbs are comprised largely of swollen leaf bases, some of which terminate in bladed leaves and some of which do not, that are attached to a highly compressed stem. Shapes range from globular to round to ovate to flat; presumably due to the growth patterns of the swollen leaf bases (**Figure 3**).

### 1.3 Tomato and pepper

The wild tomato species *Solanum pimpinellifolium* possesses fruits that are round and weigh approximately 1 g, whereas fruits from *S. lycopersicum* var. *cerasiforme, the* progenitor type from which tomatoes were domesticated, weigh 10–30 g and may possess oval or flat shapes in addition to the traditional round wild-type shape. Cultivated tomato fruits may reach 1 kg and beyond, and are found in a wide variety of shapes. Tomatoes have likewise been selected for a variety of fruit shapes, including globe, blocky, flattened, elongated, pear, heart, round, and cylindrical types (Sacco et al., 2015), or visually classified into eight fruit shape categories: flat, round, rectangular, ellipsoid, heart, long, obovoid, and oxheart (Rodriguez et al., 2010). References to shape in tomato go back at least as early as the 19^th^ century. The *Statistica murattiana* (1811), a statistical report of agriculture and trade by mathematician Napoleone Sinibaldo Piaggio, reports that tomatoes were cultivated in a variety of shapes on the outskirts of Naples. Another agricultural treatise of the same year, *L’ortolano dirozzato* describes the shape of the varieties grown: *schiacciato* (squashed or flattened), *globoso* (spherical), and *peretto* (pear shape). This is likely the first mention of the pear-shaped tomato (Gentilcore, 2010), which became very popular in Italy and beyond.

Although round tomato fruits are in heavy demand globally, it is a given that humans also display preferences for non-wild-type shaped fruit. The Plant Genetic Resources Unit (PGRU) of the United States Department of Agriculture (USDA) has collected and preserved more than 6,600 accessions of tomato and its wild relatives. According to the morphological descriptors for fruit shape that have been characterized in about half of the collection, the predominant classes are slightly flattened, round, flat, high rounded, long, blocky, plum, pear, long oblong, and heart (**Table 1**). Only approximately 20% of these accessions display the round wild-type fruit shape. Data from the Economic Research Service (ERS) of the USDA indicate that tomatoes with round, plum/roma, or oval shapes are the predominant fruit shapes found in import and export markets for fresh tomatoes over the last several years. Notable among these data are the profusion of accessions and market classes that feature non wild-type fruits.

**Table 1:** Primary fruit shapes among 2,979 accessions of tomato and its wild relatives in the USDA-NPGS collection.

Tomato Fruit Shape	Number of Accessions
SLIGHTLY FLATTENED	771
ROUND	599
FLAT	477
OTHER	396
HIGH ROUNDED	225
LONG	141
BLOCKY	109
PLUM	100
PEAR	78
LONG OBLONG	59
HEART	24
*Total*	2979

Five distinct species of domesticated peppers exist, which were developed from wild species native to the Americas (Cao et al., 2022). Wild pepper fruits were erect, in contrast to domesticated fruits, which are pendent. Pendent fruit position allows for greater fruit size and protection from both predators and sun (Paran and van der Knaap, 2007). Selection for larger, blocky-shaped fruit took place more recently, with evidence of sweet-fruited types emerging in the last several centuries. The greatest variation in fruit shape and size occurs in the species *Capsicum* annuum. Some examples from this species include large, non-pungent, blocky-fruited types such as the bell pepper; poblano or ancho peppers with very dark green fruit and concave shoulders, rich flavors, and moderate pungency; long, slender, mild Anaheim or NuMex types used for roasting and for fresh eating; cayenne types, which are very thin and often ground into powder, to be used as an ingredient in soup, chili, and other dishes; Jalapeno types, which are small and dark green and a common ingredient in Mexican cuisine; pimiento or cherry types, which are small, red and round; and banana types, which are long, mild, tapered, and possess waxy fruit. Habanero types, which come from the species *Capcisum chinense*, are extremely pungent and have small, dimpled fruits. The species *Capscium baccatum* contains Aji peppers, which are smaller and more berry like. The species *Capsicum frutescens* contains the tabasco pepper, which has small, pendent fruit and is quite pungent.

### 1.4 Melon and watermelon

Wild relatives of melon *(Cucumis melo)* exhibit a range of phenotypic diversity in southern Asia, Australia, Africa, and India. Recent studies suggest that the crop may have been domesticated multiple times in both Africa and Asia (Endl et al., 2018). Wild melon fruits are round and typically weigh less than 50 g (Monforte et al., 2014). The initial domesticates may have been selected primarily for their lipid and protein-rich seeds, rather than for their fruits. Selection for an expanded mesocarp, resulting in fleshy domesticated melons, took place later. This diversifying selection developed dozens of melon types, including fruits that weigh more than 10 kg. Some of the primary melon types include *cantaloupensis*, which have deeply grooved exteriors and hard, scaly rinds; *reticulatus*, which have netted exteriors; *inodorous*, which can be round to oval and include fruits that have pointy shapes; and *flexuosus*, which is also known as the snake melon and may have slender, cucumber-like fruit. Western shipping melons, which are often referred to as cantaloupe, are round to oval in shape and typically netted and without sutures on the exterior. Eastern melons are round to overall, netted, and typically sutured. Casaba melons may be acorn-shaped with bright yellow exteriors. Honey Dew melons are round to oval with green-white exteriors. Crenshaw and canary melons have elongated acorn-shaped fruits with flattened stem ends. Charentais melons are globe to elongated in shape with netted or smooth exteriors. Mediterranean, Hami, and Rochet types have oval fruits, although there are Hami types that exhibit globe-shaped fruits. Persian and Japanese types have round to slightly oval fruits.

Watermelon belongs to the genus *Citrullus*. In addition to *C. lanatus*, the sweet watermelon consumed around the world, the genus also contains *C. mucosospermus, C. amarus, C. colocynthis, C. rehmii, C. ecirrhosus and C. naudinianus* (Chomicki and Renner, 2015). Northeast Africa, specifically the Darfur region of Sudan, is the likely region of domestication for the crop (Paris, 2015; Renner et al., 2017; Guo et al., 2019). Fruits may be round, oval, elongate, or spherical, and the fruit’s surface may be sutured or smooth. Recently, small-fruited types have surged in popularity in certain markets, but round and elongated melons remain popular in many world regions.

## 2 Assessment of organ shape in vegetable crops

Market class differences may represent subtle shifts in contour and shape, which have historically been classified visually. Because of the subjectivity of such assessments, precision is often lacking in describing differences in shape among representatives of different market classes. For example, European Union marketing standards for tomato list four market classes: round, ribbed, elongated/oblong, and cherry/cocktail (Commission implementing regulation (EU), 2011). No specific guidelines for the fruit shape in these market classes are provided, however the regulations do specify allowable size variation within a particular market class for marketing purposes. Regulations also describe restrictions and permission on certain aspects of shape such as “no excessive protuberances” and the allowability of a “small umbilicus,” but few specific details on shape *per se* are prescribed. United States marketing standards are similar in that no particular shape parameters are offered for tomato and only size grades must be marked. Furthermore, “Cerasiforme types” (cherry) and “Pyriforme types” (pear shaped) are exempt from marketing requirements entirely (United States Standards for Grades of Fresh Tomatoes, 1991). A similar situation exists for carrot, where the U.S. marketing requirements specify size grades but do not regulate shape (United States Standards for Grades of Carrot, 2020). Despite the lack of standardization in the commercial realm, tomatoes are classified scientifically based on contour points, followed by elliptical Fourier modeling (Visa et al., 2014). This type of classification allowed for the detection of 9 shape categories in tomato that are also discernable by growers. The contour measurements shape categories are round, rectangular, ellipsoid, flat, obovoid, oxheart, long rectangular, heart and long (Visa et al., 2014).

Carrot can be classified into a range of market classes, including Imperator/Cut-and-Peel (longest type), Danvers and Chantenay (large U.S. processing types), Nantes (fresh market type), Parisienne (shortest type), Amsterdam, Kuroda, Flakee, Belgian and Berlicum (Simon, 2000) and more. The difference among many of these types might be found in the characteristics of the shoulders and tips. For instance, Nantes and Danvers might differ in the tip, where Nantes types of exhibit tips with a greater degree of bluntness, and in the shoulder, where Danvers may have greater shoulder width. Imperator and Parisienne exhibit large differences in length, where Imperator types are used for “cut and peel” carrot products and Parisienne is primarily a specialty market small, round carrot. Kuroda and Chantenay both exhibit shoulders with prominent angles, but Chantenay types are typically much bulkier. Regardless of these differences, the fact that visual assessment is the only means for assignment to a market class means a greater level of subjectivity associated with shape.

Prior to the advent of digital imaging systems, hand measurements such as the ratio of crown diameter to root length, were used to assess root shape (Bradley et al., 1967). Bleasdale and Thompson (1963) proposed cylindricality (*C*), as defined as


$$C=\frac{w}{\pi r^{2}l}$$

where *w* is root fresh weight, *r* is the radius of the crown and *l* is the root length. The values of *C* lie between 0.33 for a cone and 1.0 for a cylinder. They also proposed that length of the roots was proportional to the logarithm of crown diameter. They further proposed two simple relationships: (i) that logarithm of diameter is linearly related to logarithm of weight; and (ii) length is linearly related to diameter. These relationships provided realistic predictions of yields of carrot and beet in diameter grades from observed weights in independent datasets. Stanhill (1977) reviewed the factors that influenced carrot shape. He summarized that besides large genetic-based differences, roots become more cylindrical with increasing plant density, at air temperatures below 18°C, in drier soils, when the shoots have been defoliated and when the plants are younger. Benjamin (1987) speculated that the shape of the storage root might relate to the spatial pattern of cross-linking of hydroxyproline-rich glycoproteins of cell walls (Stafstrom and Staehelin, 1988). Luby et al. (2016) found that while carrot market classes demonstrate some phenotypic and some genetic differences, they are largely a construct of breeders and are therefore malleable. For example, while cultivars from a single market class are more genetically similar than cultivars from different classes, principal component analysis of genotyping by sequencing (GBS) data showed that the first two principal components explained only 10% of the total variation among a collection of 140 US carrot cultivars, and only 12.5% of the variation within the USDA-Plant Introduction collection of carrot accessions collected from around the world.

Plant phenotyping has advanced substantially in the past several decades, and digital imaging has facilitated sophisticated approaches to contour and shape analysis of plant organs (Horgan et al., 2001; Brewer et al., 2007; Hameed et al., 2018; Brainard et al., 2021). Such approaches have made shape quantification quite precise. Imaging platforms allow for the quantification of contours such as shoulder and tip curvature in roots or the roundness of fruits that are not simply the product of length and width (Iwata et al., 1998; Miller et al., 2017; Turner et al., 2018). Other traits such as distal and proximal angles, and various degrees of obovoid, round and ellipsoid can now be quantified as well (Brewer et al., 2006; Rodriguez et al., 2010). Typically, these attributes are more intuitive to growers than analyses based on contour measurements.

Brainard et al. (2021) demonstrated a digital imaging platform for carrot that involves a three-stage workflow: image acquisition, image pre-processing, and image analysis. Images are staged to include a QR matrix barcode that contains identifying information for the sample as well as a scale bar for converting pixel measurements to physical distances. Images are acquired with a digital camera, and processed using a set of custom Python scripts which first convert RGB images to grayscale. These images are then smoothed and used to generate binary masks; which are black and white images in which the white pixels indicate the plant organ and black pixels the background. The tip of the root is identified through an algorithm that identifies points of maximum curvature along the root contour. A straightening procedure was then developed that uses Euclidean distance transformation to trace a root’s midline from the tip to the top of the root, and then samples the binary mask along vectors normal to the tangent of this midline. Curvature values of the straightened root are estimated by fitting splines to segments of the contour. Root size is measured as the total area of the binary mask. Tip angle is measured as the interior angle formed by the line segments connecting the tip of the carrot to contour points located 10% up the length of the carrot toward its top, while shoulder hull area is the area encompassed by background pixels in the rectangular region bounding the top 10% of the carrot. Principal component analysis of the root profile is also utilized to provide more agnostic measures of variability in shape and size.

Other approaches to assessing the shape of plant organs have been developed. Turner et al. (2018) developed a digital imaging platform for quantifying shoot and root shapes in carrot. They developed binary masks that were evaluated in a manner similar to that of Brainard et al. (2022). The software tool they developed is available through the CyVerse Discovery Environment web interface. This platform allowed for the assessment of shoot height, root length, root width, convex hull, eccentricity, equivalent diameter, Euler number, perimeter, solidity, petiole width, petiole number, and petiole length. Turner et al. used this platform to identify numerous quantitative trait loci (QTL) for shoot and root traits in carrot. Paulus (2019) pioneered the use of 3D sensing to image plant organs. Paulus describes techniques like laser triangulation, time-of-fight, terrestrial laser scanning for measuring traits like leaf width and length, plant size, and characteristics of plant organs. One approach with this platform is to use machine learning for shape assessment. Miller et al. (2017) developed a high-throughput method for assessing maize ear and kernel attributes from digital images. In their platform, an algorithm determines the average space each kernel occupies along the cob axis using a sliding-window Fourier transform analysis, while a second counts individual kernels. Another algorithm assesses the axes of each kernel following a Bayesian analysis of contour points, which finds the tip of the kernel. Ear and shape traits are then assessed via principal component analysis of contour points.

Elliptic Fourier Descriptors have also been developed as a tool for capturing and analyzing complex shape characteristics of plant organs. This technique employs a mathematical function to decompose curvatures into coefficients, representing frequencies or harmonics. These coefficients provide quantitative representations of shape, which can be further analyzed using principal component analysis. Elliptic Fourier Descriptors have been successfully applied to measure the shape of plant organs in various crops, including tomato, strawberry, radish, soybean, and buckwheat (Furuta et al., 1995; Iwata et al., 1998; Ohsawa et al., 1998; Visa et al., 2014; Nagamatsu et al., 2021).

Van der Knaap and colleagues developed a digital tool called Tomato Analyzer for analyzing the shape of fruits, including flat, ellipsoid, rectangular, oxheart, heart, long, obovoid, and round (Darrigues et al., 2008; Gonzalo and van der Knaap, 2008; Gonzalo et al., 2009; Rodriguez et al., 2010). The software automatically quantifies 37 shape attributes with unique mathematical descriptors. This platform is capable of assessing eccentricity, which relates to the position of the seed cavity inside of the fruit; asymmetry in fruit shape, which relates to the degree to which the fruit is top or bottom heavy; latitudinal section, which relates to the degree of uneven shape of the fruit or lobedness; thickness of the pericarp, septum, and placenta; and distal end protrusion and proximal end angle, which relate to the area of the protruded end of the fruit and the angle of the proximal end, respectively. Five different progeny populations derived from crosses between cultivated tomato *Solanum lycopersicum* and the wild relative *Solanum pimpinellifolium* were analyzed for fruit shape using this platform and numerous shape QTL distributed across the tomato genome were identified (Brewer et al., 2007; Gonzalo and van der Knaap, 2008). The platform has since been used by numerous researchers throughout the world to assess shape characteristics in horticultural crops.

## 3 Genetic and molecular mechanisms underlying shape and size

**Figure 4:**
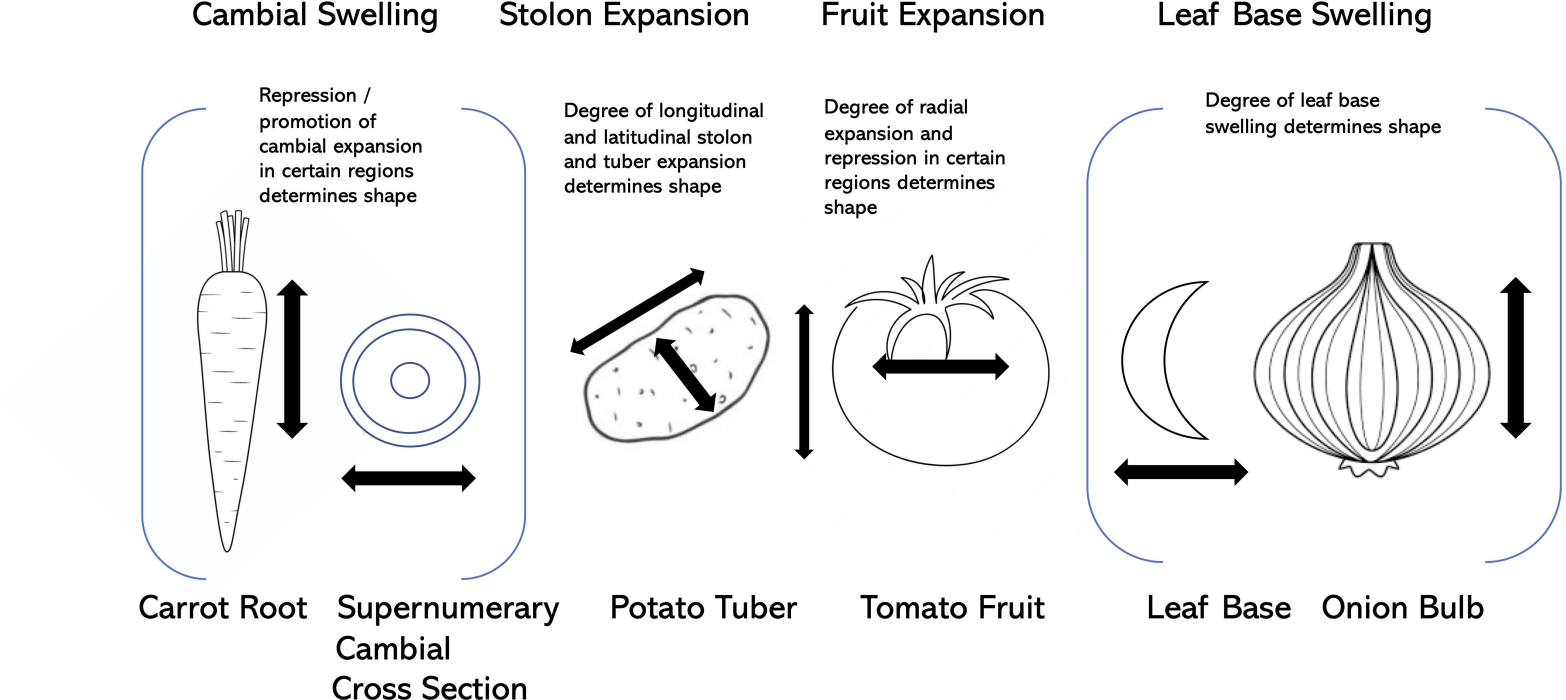
Modes of expansion and shape formation in carrot, potato, tomato, and onion.

As laid out above, the selection for visually-pleasing shapes in vegetable crop fruits, roots, leaves, stems, tubers, and other organs has taken advantage of a number of opportunistic factors, including modification of supernumerary cambia, allelic variation at loci that control fundamental processes such as cell division and cell expansion, hormonal regulation, and partitioning of photosynthate (**Figure 4**). Some of the key genes associated with these shape modifications are presented in **Table 2**.

**Table 2:** Examples of genes and proteins with influence on organ shape in vegetable crops.

Plant species	Gene or protein	Effect	Reference
Fruit			
*Solanum lycopersicon*	*ovate*	Elongated fruit, pear shaped fruit	Liu et al., 2002
*Solanum lycopersicon, Cucumis melo, Solanum tuberosum*	OVATE Family Protein-20, *OFP20, OFP13*	Obovoid shaped tomato and melon fruit when combined with *ovate*, root shape regulation in radish, altered tuber shape from round to oval	Wu et al., 2018; Wang et al., 2022a
*Solanum lycopersicon, Cucumis melo, Capsicum annuum, Oryza sativa*	*TRM, TRM5, TRM25, GW7*	Interact with OFP, rescues obovoid shape	Wu et al., 2018
*Solanum lycopersicon, Citrullus lunatus, Cucumis melo, Cucumis sativus, Oryza sativa*	*SUN, Cla011257, Csa1G575000, CmFS2.1, IQD21*	Elongated fruit shape, oxheart fruit shape	Xiao et al., 2008; Liu et al., 2014; Pan et al., 2017; Dou et al., 2018
*Solanum lycopersicon*	ERECTA	Blocky fruit shape	Sun et al., 2015
Root and tubers			
*Ipomea batatas*	*KNOX genes SRF1*, *SRF5*, *SRF6*	Root development	Ravi et al., 2014; Tanaka et al., 2008
*Ipomea batatas*	*IbEXP1*	Root thickening	Noh et al., 2013
*Solanum lycopersicon*	*LONELY GUY 1*	Promotes minituber / storage organ formation	Eviatar-Ribak et al., 2013
*Solanum tuberosum*	*StCDF1*	Tuber development	Kloosterman et al., 2013
*Daucus carota*	*DCv3_Chr5.21023*, *terminal ear1*	Root development	Wang et al., 2018; Mousavi et al., 2021; Brainard et al., 2022
*Brassica rapa*	*Bra-CYP735A2*	Root-hypocotyl development	Liu et al., 2019
*Raphanus sativus*	RsOFP2.3	Involvement in root shape	Wang et al., 2020a.
Bulbs			
*Allium cepa*	*AcFT1, AcFT4*	Bulb development	Ar Rashid et al., 2019

### 3.1 Tomato

OVATE is the founding member of the OVATE Family Proteins (OFPs) class, and encodes a negative regulator of growth which reduces fruit length (Liu et al., 2002; Hackbusch et al., 2005; Wang et al., 2007). Pyriform fruit in tomato, which is associated with an obovoid shape, was shown to be associated with a single recessive allele more than 100 years ago (Hedrick and Booth, 1907). Oval-shaped fruit co-segregated with pear-shaped fruit, and this locus eventually became known as ovate, *O*. Liu et al. (2002) identified a SNP in one of the ORFs which was associated with an early stop codon in *ovate*, and a 75-amino acid truncation in the C-terminus of the predicted protein. A conserved domain in the *OVATE* gene was largely eliminated by this truncation.

Today, it is recognized that many plants contain OFPs and that these OFPs have multiple roles in plant development, in particular in organ shape not only in tomato but also in pepper, melon and potato (Liu et al., 2002; Wu et al., 2018; Lee et al., 2020; Borovsky et al., 2022; Martínez-Martínez et al., 2022). For example, a major QTL associated with fruit shape index and distal fruit end angle in pepper contains an ortholog of SlOFP20 (Borovsky et al., 2022; Lopez-Moreno et al., 2023). In tomato, the *ovate* mutation is not associated with a single type of change in fruit shape, but instead it appears that *ovate* interacts with modifiers to produce an array of fruit shapes (Rodriguez et al., 2013; Wang et al., 2016a; Wu et al., 2018).

The *ovate* allele is a result of a premature stop codon and relieves the inhibition on fruit growth resulting in elongated fruits (Liu et al., 2002). The naturally occurring *ovate* mutation is found in elongated tomato accessions with obovoid/pear-shaped fruits, as well as rectangular, ellipsoid and heart shaped fruits. The OVATE gene was cloned from the *S. lycopersicum* subspecies cerasiforme variety ‘Yellow Pear’, which implies that this mutation arose in the progenitor species and was maintained in the cultivated germplasm pool (Paran and van der Knaap, 2007; Rodríguez et al., 2011).

Another fruit shape gene originally found in tomato is *SUN*, encoding one of the members of the plant-specific gene families *IQ67 domain protein*/*SUN-like* (*IQD/SUN*). In tomato, the *SUN* locus contains a QTL that was originally revealed from a cross between the wild round-fruited *S. pimpinellifolium* and the cultivar ‘Sun1642’, which exhibits elongated fruit (Xiao et al., 2008). A 25 kb insertion present in ‘Sun1642’ but not in the wild species is associated with this fruit shape QTL. The locus arose from an interchromosomal gene duplication event mediated by a retrotransposon, *Rider*. This insertion provided an opportunity for increasing the expression of the gene relative to the ancestral version, leading to elongated fruit shape. In this case, the mutation resulted in a gain-of-function event. Subsequently, *SUN* family members have been shown in other crops to underlie shape variation such as rice, cucumber and watermelon (Duan et al., 2017; Pan et al., 2017; Dou et al., 2018; Legendre et al., 2020). Thus, like OFPs, SUN members are also often associated with produce shape in agricultural crops.

A highly conserved set of genes across plant species are *WUSCHEL* and *CLAVATA3*. IN tomato, fruit locule number and flat shape is controlled by *FASCIATED* (*FAS*, ie, *CLAVATA3;* Barrero and Tanksley, 2004; Xu et al., 2015) and *LOCULE NUMBER* (*LC*, ie, WUSCHEL). These genes control meristem organization and when mis-expressed, larger fruits ensue. None of the wild relatives of tomato carry mutations in these genes with the exception of *lc* (Blanca et al., 2015; Rodríguez et al., 2011; Wu et al., 2018). The *LC*, *FAS*, and *SUN* mutations appear to have arisen in the same ancestral population, while the *OVATE* mutation arose in a separate lineage (Rodriguez et al., 2010). For as long as humans have been selecting crops, such traits reflect human desires and preferences rather than those influenced only by natural selection.

Another gene that controls shape are members of the TONNEAU1 Recruiting Motif (TRM) family. TRMs interact with TONNEAU1 (TON1) which is a plant-specific protein that nevertheless shares protein domains with the animal centrosomal proteins (Camilleri et al., 2002; Azimzadeh et al., 2008). FASS/TON2 encodes the regulatory subunit of a Protein Phosphatase2A, and together with TON1 and TRM proteins forms the TTP (TON1-TRM-PP2A) complex (Spinner et al., 2013). TRMs recruit TON1 to the cytoskeleton through the M2 domain (Drevensek et al., 2012) and the TTP complex is required for the formation of the PPB and cell division (Spinner et al., 2013). Many members of the TRM protein family decorate microtubules in *Nicotiana benthamiana* cells (Drevensek et al., 2012; Wu et al., 2018; Zhang et al., 2023). IQD/SUNs interact with microtubules (Bürstenbinder et al., 2013; Bürstenbinder et al., 2017; Wendrich et al., 2018; Yang et al., 2019, Li et al., 2021) and regulate cell division pattern for organ shape regulation probably by facilitating PPB formation and division-plane orientation (Kumari et al., 2021). In tomato, OVATE interacts with a subset of the TRM family that contains the M8 motif and functions in organ shape regulation (Wu et al., 2018; Zhang et al., 2023). A knockout mutation in *TRM5* partially rescued the elongated fruit shape caused by *ovate* and *ofp20* (*ovate/sov1*) (Wu et al., 2018), while mutation in *SlTRM19* further elongates the fruit shape in *ovate/sov1* (Zhang et al., 2023). *SUN/IQD* also genetically interact with *OVATE*, *OFP20*, and *TRM5* in tomato fruit shape regulation (van der Knaap et al., 2014; Snouffer et al., 2020). What is less well understood is how OFPs complicate the interaction of TRMs and IQD/SUNs at the microtubules, and the resulting effect on plant organ shape. Subcellularly, co-expression of tomato TRM5 and OVATE results in re-localization of TRM5 from the microtubules to the cytosol in *Nicotiana benthamiana* cells. On the other hand, co-expression of tomato TRM5 and OFP20 results in relocalization of OFP20 from the cytosol to the microtubules, and their interaction is OFP and M8 domains dependent as well as gene co-expression dependent (Wu et al., 2018; Zhang et al., 2023). These results suggest that specific OFP-TRM interaction may dictate the localization of the TTP complex and microtubule organization. Snouffer et al. (2020) have proposed a model for the interaction among OFPs, TRMs and IQDs in microtubule organization to impact cell division and, ultimately, organ shape. The expression of different OFPs over developmental time and space may also serve to coordinate cellular response and microtubule organization during organ outgrowth. Similar to OFP and SUNs, TRMs are also found to regulate shape in other crops such as pepper, rice and cucumber (Wang et al., 2015c; Wang et al., 2015d; Wu et al., 2018; Taitano, 2020; Xie et al., 2023).

### 3.2 Sweet potato and potato

Sweet potato is currently among the best models for the study of storage root formation. The cultivated sweet potato evolved from the wild tetraploid *I. trifida* and diploid *I. trifida/I. tabascana* species, which do not form storage roots (Ponniah et al., 2015). In sweet potato, the differentiation of vascular cambia causes cell division and expansion of parenchyma cells for storage of starch granules, which leads to rapid bulking and starchy root formation. Theory suggests that storage root initiation is influenced by cambium propagation and lignification. Three class I *knotted*-like homeobox (*KNOX1*) genes—*SRF1*, *SRF5*, and *SRF6* modulate carbohydrate metabolism and cell division in sweet potato and play a primary role in storage root development (Ravi et al., 2014). Tanaka et al. (2008) suggested that *KNOX1* genes may regulate cytokinin levels and therefore be involved in storage root development.

Several genes have been identified as controlling tuber formation in potato. Potato was domesticated in the Andes and adaptation to long days in Europe, North America, and parts of South America was associated with regulation at the *StCDF1* locus. This locus affects tuberization via *CONSTANS (* Abelenda et al., 2016 *)*. Truncated alleles at this locus, mediated by transposable elements, deregulate the circadian clock and allow for tuberization under long days. Hardigan et al. (2017) found truncated alleles of *StCDF1* from wild species were introgressed into adapted long-day cultivars at the main locus controlling maturity. Pandey et al. (2022) genotyped a diversity panel of 214 advanced clones and phenotyped it in three field environments in Texas. A genome wide association study (GWAS) revealed QTL for tuber shape and eye depth on chromosomes 5 and 10, one of which was located near this same *StCDF1* locus.

Another important aspect of their findings concerns QTL for cell cycle and endoreduplication effects. Endoreduplication is associated with an absence of cytokinesis and successive rounds of DNA replication. Ploidy levels increase, along with cell size and volume. Endoreduplication has been associated with other storage tissues in domesticated species, such as maize. In this case, it appears that genes associated with modifications to the cell cycle may have been important regulators of size and shape of potato tubers. Hardigan et al. (2017) suggest that selection of endoreduplication promoting alleles during domestication may have been important contributors to size differences in domesticated potato.

Potato tubers vary in shape from flat/compressed to spindle shaped/elongated, and substantial variation exists in the wild diploid relatives of cultivated potato (Fan et al., 2022). Shape behaves as a quantitative trait, with the *Ro* locus on chromosome 10 as one of the primary QTL associated with shape traits. The potato tuber shape gene is controlled by the ortholog of tomato OFP20, located in the same syntenic region of the genome as tomato (Wu et al., 2018). Round is dominant to elongated when genotypes at the *Ro* locus alone are considered. Huang et al. (2022) identified a novel QTL for tuber shape in a population derived from a wild species, So*lanum chacoense*. They identified a QTL, *TScha6*, for tuber shape on chromosome 6. Four SSR markers and 20 CAPS markers near this QTL were used to screen a set of cultivars and breeding lines. One of the markers, C6-58.27_665, was significantly associated with tuber length/width ratio. The authors proposed the use of this marker in breeding for shape traits in potato.

*StOFP20* has recently been shown to have a function in tuber shape. In potato, *StOPF20* is highly expressed in the tuber initiation stage. Null mutation in *StOFP20* turns round tuber shape into oval (Ju et al., 2023). Similar to tomato, StOFP20 can directly interact with three TRM proteins (StTRM5, StTRM19 and StTRM20), which was shown by yeast two-hybrid (Y2H) and firefly luciferase complementation assays (Ju et al, 2023). Engineering *StOFP20* by modulating gene expression level also causes altered tuber shape in several potato varieties (Van Eyck et al., 2022).

### 3.3 Beet, carrot, parsnip, and turnip

Beet, carrot, parsnip, and turnip are all examples of biennial root crops domesticated from annual species, all of which had non-succulent roots, in an effort to produce a succulent vegetable. The vegetable in each case is largely composed of supernumerary cambia, possessing additional swollen and expanded xylem and phloem tissues beyond their primary xylem and phloem. It is this unique feature that allows for their girth and therefore their success as vegetables (Goldman, 2020). Photosynthate produced in the leaves and destined for fibrous roots and the shoot apical meristem are translocated via the storage organ. Similarly, water and nutrients are translocated from the fibrous roots to the storage root and eventually to the leaves. In species without swollen storage organs, photosynthate, water, and nutrients are transferred directly from root to stem. It is possible that the carbohydrates contained by underground storage organs were important energy sources during hominid evolution when other foods were scarce (Abelenda and Prat, 2013), which would have driven the domestication and evolution of root and tuber crops.

One of the primary differences associated with the contrast between fruit and root shape is related to cell division. Storage roots are characterized by cell division and expansion throughout their development (Milford, 1973; Ting and Wren, 1980; Hole et al., 1984; Benjamin, 1987). In contrast, fruit storage organs are characterized by an initial phase predominated by cell division, followed by a phase of cell expansion with no cell division. The storage organs of all four of these root crops develop by the formation of supernumerary cambia. In all four of these species, there is no distinct limit to the distance down the tap root these cambia can extend.

The storage of carbohydrate in these roots is made possible by storage parenchyma cells that are living, allowing the root to accumulate carbohydrate throughout its growth period. In all storage root crops like beet, carrot, parsnip, and turnip, the storage parenchyma cells are produced by secondary tissues, which arise from division of the vascular cambium. We might refer to these growth processes as anomalous growth, or growth that does not follow recognizable patterns that occur commonly in the majority of vascular plants. Growth in the girth of beet, carrot, parsnip, and turnip is due to meristematic activity in the vascular cambium, producing xylem on the inner side and phloem on the outer side of the stem (Robert et al., 2011). Robert et al. (2011) argue that growth via successive cambia provides an advantage to plants under water stressed conditions, and that species with successive cambia were common in drought or salt conditions.

More than 75 plant genera form successive cambial layers, each of which produce secondary xylem and phloem (Spicer and Groover, 2010). Wild relatives of the root/hypocotyl crops beet, carrot, parsnip, and turnip exhibit at least one additional cambial layer beyond the primary xylem and phloem, and the existence of these additional tissue layers were likely a key reason for the success of the domesticated forms. These forms exhibit growth along both a vertical plane perpendicular to the soil and a horizontal plane parallel with the soil. The relative degree of vertical and horizontal growth, as well as the portions of these cambial strips that swell, are some of the primary determinants of root/hypocotyl shape.

Among certain species in the genus *Beta*, the first cambial layer forms between the primary xylem and phloem and produces secondary xylem and phloem. The second cambial layer forms from the parenchyma tissue within the stem cortex and produces conjunctive tissue to the inside. Sometimes this cambial tissue produces secondary xylem and phloem directly, with new cambia coming from the oldest phloem tissue. Sometimes it functions as a master cambium, producing conjunctive tissue and new cambia on the inside. Regardless, there are repeating increments of secondary xylem and phloem in between conjunctive tissue. These repeating layers of supernumerary cambia are clearly apparent in both the wild relative, *Beta vulgaris* subsp. *maritima*, and cultivated table beet, *Beta vulgaris* (**Figure 2**). These tissues are of functional significance because the additional parenchyma can assist with both carbohydrate and water storage. The existence of these layers in wild predecessors allowed for relatively simple human selection for swelling and expansion, giving rise to the many root shape variants of cultivated beets today (**Figure 2**).

In carrot and parsnip, the vascular cambium produces a large amount of storage parenchyma in its secondary phloem tissue. This is where carbohydrates will be stored. This tissue also contains normal conducting cells typical of phloem, but they represent a small amount of this secondary phloem. Beet forms many concentric cambial layers, each of which produces xylem inwardly and phloem outwardly. Groups of lignified cells in the phloem give rise to the zoning or rings typical in a cross section of a beet root (Forbes and Watson, 1996). The red-pigmented rings consist of storage parenchyma, whereas the lighter colored rings are composed of xylem and phloem (**Figure 2**). The alternation of the lighter and darker bands shows how successive cambia represent a successful mechanism for the interspersing of vascular tissue (which input and remove stored sugars) between cylinders of storage tissue. In turnip, a small amount of secondary phloem is formed initially, but the secondary cambia in the xylem parenchyma divide to give rise to secondary phloem that are scattered through the xylem, and this helps distribute carbohydrate throughout the vegetable (Forbes and Watson, 1996).

Many key transcriptional regulators of developmental processes associated with the shoot apical meristem are also expressed in the cambial zone during secondary growth (Schrader et al., 2004). Groover (2005) hypothesized that genes involved in shoot apical meristem growth were co-opted during the evolution of cambia and secondary vascular growth. Despite the potential source for the origin of these genes, there are fundamental differences between the radially organized cambial zone and the three-dimensional organization of the shoot apical meristem.

Brainard et al. (2022) used a digital imaging system to perform a GWAS and calculated genomic-estimated breeding values (GEBVs) in segregating populations of carrot that had been developed by crossing parents from different market classes that possessed different shapes. They showed that the components of market class -and thus root shape- are polygenic traits, likely under the influence of many small effect QTL. High predictive ability of GEBVs were noted, reflecting high levels of additive genetic variance for shape traits. Multiple QTL were identified for length, aspect ratio, maximum width, and root fill. Root fill was described as a size-independent aspect of carrot root shape referring to the degree of maintenance of width along the length of the carrot root. In this sense, root fill is related to yield and biomass (Brainard et al., 2021). The root fill QTL was the first genetic characterization of the control of the vast majority of the variance in carrot root shape. In the diversity panel they used, QTL with relatively small effects were detected for some of the other shape parameters, although heritabilities associated with these traits were calculated as relatively high (Turner et al., 2018; Brainard et al., 2021). It is possible that selection for root shape occurred for numerous accessions in the diversity panel they studied, potentially complicating the search for common QTL controlling shape.

Brainard et al. (2022) reported several candidate genes for key QTL associated with shape in carrot, including the gene of DCv3_Chr5.21023, which has previously been shown to play a role in root development. This gene is a predicted piezo-type mechanosensitive ion channel, which may be associated with the ability of roots to penetrate through the soil profile (Mousavi et al., 2021). The DCv3_Chr9.36166 gene was found in region of a QTL associated with aspect ratio. This gene is a homolog of protein terminal ear1, which has been associated with abscisic acid-mediated root growth (Wang et al., 2018; Brainard et al., 2022). Three genes were found in linkage disequilibrium with a QTL on chromosome 2 associated with root fill. DCv3_Chr2.08059 encodes a homodomain-leucine zipper (HD-Zip) protein that has been linked to root development (Elhiti and Stasolla, 2009). DCv3_Chr2.08061 is a homolog of non-DNA-binding bHLH transcription factors that are involved in lateral root formation (Castelain et al., 2012). DCv3_Chr2.08063 is homologous to a AAA-ATPase protein found in *Arabidopsis thaliana* that has been found to drive adventitious root formation (Xu et al., 2018).

Wang et al. (2020a) found that *RsOFP2.3* was the top candidate gene for the involvement of tuberous root shape in radish as its expression was negatively correlated with tuberous root elongation after the cortex splitting stage, and ectopic overexpression of the gene in *Arabidopsis* led to shorter but wider hypocotyl and siliques. This gene is another example of an OFP that regulates organ shape. Mitsui et al. (2015) performed transcriptomic analysis in radish that revealed genes in carbohydrate metabolism played an important role in root thickening.

Turnip is often considered a root crop, but the storage organ that comprises the crop is actually a hypocotyl plus a compressed stem and a root. It is, however, possible to see leaf scars toward the tops of the turnip (Liu et al., 2019), which can allow for the development of side shoots and to some extent parallels the situation in tuberous crops that are formed from underground stolons. Liu et al. (2019) have identified QTL for the production of side shoots in turnip. They have also pointed out the potential for adventitious root formation on the bottom of the root, which can result in a forked or fanged like appearance. They use the descriptor “hypocotyl-tuber” to more accurately describe the turnip. Liu et al. (2019) found that morphological changes occurred in the xylem of turnips 16 days after sowing that were predictive of eventual hypocotyl/root size and shape. The *Bra-FLOR1* paralogue exhibited increased expression 16 days after sowing, at the point when the hypocotyl starts swelling. Since this gene is associated with flowering, the authors suspected a possible dual role of this gene in both reproductive growth and hypocotyl/root formation. The *Bra-CYP735A2* gene was identified for its possible role in hypocotyl/root growth via trans-zeatin. Wu et al. (2021) crossed turnip with Chinese cabbage and identified QTL for swollen rootedness. Two of these QTL, FR1.1 and FR7.21, were confirmed in multiple populations. QTL FR7.1 was circumscribed to a 220 kb region containing 47 putative genes, one of which, Bra003652, is a homolog of AT1G78240 that plays a role in cell adhesion and tumor-like formation in *Arabidopsis thaliana*.

Iwata et al. (1998) used elliptic Fourier descriptors to describe radish root shape. The coefficients of these Fourier descriptors were associated with the shape characteristics of market classes for radish, and could be used for breeding shape related traits. Iwata et al. (2004) conducted a diallel analysis on root shape parameters measured over time in radish and concluded that length to width ratio is predictable at a very early growth stage, whereas tip bluntness and roundness of the middle portion of the root may only be accurately selected at harvest time. Wei et al. (2022) describe that the root shape of radish is measured with the ration of root length to root diameter at the maximum root diameter. The diameter of radish roots and the length of radish roots range from 1 to 30 cm and 3 to 200 cm, respectively. Radish roots are divided into 15 main shapes, including cylindrical, conical, oval, oblate, pear-shaped and round. Many QTL have been identified as responsible for variation in root shape. Wei et al. (2022) found seven QTL for root shape on five chromosomes (R1, R2, R4, R5 andR7).

### 3.4 Onion and related Alliums: carbohydrate storage in leaf bases

The onion bulb is comprised of overlapping swollen leaf bases that have accumulated water and carbohydrates as a result of the bulbing process. The stem upon which leaves are formed is compressed, but it expands radially to accommodate new leaves and roots forming during the early stages of plant growth (Brewster, 1990; Goldman, 2023b). At the top of the stem, a region of cell division known as the primary thickening meristem, is formed around the apical meristem. These cells contain starch granules. As leaves are added, the bases of older leaves are pushed away from the stem apex, travel down the side of the stem, and end up near the bottom of the stem. The primary thickening meristem’s lateral growth causes the apical meristem to sink below the shoulder of the stem (Brewster, 1990). The older bases of leaves split and decay.

During the bulbing process, the youngest leaves do not form leaf blades and instead develop into bladeless leaves. In all other leaves in the onion plant except these younger leaves, the length from the base of the leaf sheath to the pore from which the next leaf develops is less than the blade length. A ratio of the blade length/sheath length of less than one is associated with bulbing (Brewster, 1990; Brewster, 1997. Because these inner leaves do not have blades, they help to form a pseudostem, which is hollow. As bulb formation progresses, the outermost leaves develop into dry, thin skins to protect the bulb. A typical onion at maturity has 2-3 dry outer skins, enclosing 4-5 swollen sheaths from bladed leaves, which enclose 3-4 swollen bladeless leaves (Brewster, 1990). At the center of the bub are small leaf initials that contain blades, which emerge when the bulb breaks dormancy and sprouts. The degree to which the bladed and bladeless leaf bases swell is a primary determinant of bulb shape (Goldman, 2023b).

Lee et al. (2013) demonstrated that *FLOWERING LOCUS T (FT)* produces a mobile protein that regulates both flowering and bulb formation. Bulb formation is regulated by two antagonistic *FT-*like genes known as *AcFT1 and AcFT4.* The former promotes the formation of bulbs while the latter prevents the up-regulation of the former and inhibits bulbing. Long-day photoperiods downregulate *AcFT4* and upregulate *AcFT1*, which results in bulb formation. It was later demonstrated that AcFT4 inhibits bulbing in short-day photoperiods. Ar Rashid et al. (2019) showed that *AcFT, AcLFY*, and *GA3ox1* genes exhibited distinctive patterns of tissue specific expression in onion, with *AcFT* genes located at sites of perception in the leaf blade, and *LFY* genes at sites of response in the leaf base, where the bulbing response occurs.

During the growth of the onion plant, cells move through the basal region of the meristem into a transition zone and then into the leaf blade. Ar Rashid et al. (2019) describe the photosynthesizing leaf blade and basal portions of the leaf as the sites of perception and response, respectively. They examined gene expression at different points along the leaf and found that the genes *AcFT1 and ACFT4* were produced in the same leaf tissue but exhibited different tissue specific expression patterns under long day or short-day photoperiods. Substantial gaps still exist in our understanding of how these genes regulate the formation of the bulb. Presumably, the greater the area of the leaf blade that swells during bulb formation, the more globe-shaped the bulb. Flatter bulbs shapes may be determined by smaller sections of leaf blade swelling in response to hormonal signals. That is, the site of response for leaf bases may vary in differentially-shaped onion cultivars. Pinpointing the specific sites responsible for swelling in leaf bases will be important in understanding how shape is regulated in onion bulbs.

### 3.5 Genetic control of hormonal regulation in root and fruit shape

Many studies have proposed that hormones play an important role in the elongation, formation, and thickening of storage roots; however, little is known about the determinants of root elongation in crops such as carrot and table beet. Roots contain a zone of cell division near the root cap, which also houses the root apical meristem. The zone of elongation of the root is located just above the area of cell division. The zone of elongation is where newly-developed cells begin to lengthen, resulting in a lengthening of the root itself. During early taproot development in sugar beet, genes involved in cell division and water and non-electrolyte small molecule transport, are preferentially expressed (Bellin et al., 2007). During the rapid growth stage of sugar beet taproots, genes controlled by hormones are up-regulated (Trebbi and McGrath, 2009). Zhang et al. (2017a) determined that several transcription factor family members were up-regulated during the rapid elongation phase of sugar beet taproot growth. In general, they found an antagonistic expression of brassinosteroid and auxin related genes during this phase; however, one of the sugar beet genotypes demonstrated up-regulation of cytokinin, auxin, and brassinosteroid signaling. Clearly, hormonal control is important in root elongation; however significant gaps exist in our knowledge of the genes associated with these processes in many root crops.

Cytokinins and auxins appear to be important in the early stages of sweet potato storage root development, while cytokinins and ABA) seem to be important in secondary thickening of these roots (Li et al., 2015; Huang et al., 2017). Eviatar-Ribak et al. (2013) found that the cytokinin biosynthesis gene *LONELY GUY 1* changes axillary meristems into aerial minitubers in tomato. Transcriptomic analysis revealed that the minitubers have an altered hormonal balance. Eviatar-Ribak et al. (2013) concluded that cytokinins may function as universal regulators of storage organ formation in plants. In the system they studied, tuber-forming potential was suppressed within the axillary meristem. Unlocking the potential for storage organ formation through genetic mechanisms such as this may provide clues regarding the formation of root and tuber crops.

Cai et al. (2022) analyzed the transcriptome of fibrous roots and tuberous roots in three developmental stages in two sweet potato varieties. They found differentially expressed genes involved in signal transduction and carbohydrate metabolism, and proposed the trihelix transcription factor (Tai6.25300) as instrumental in tuberous root enlargement. The *MADS* box gene *IbMADS1* (*Ipomoea batatas MADS-box 1*) has been implicated in hormonal regulation of tuberization in sweet potato (Ku et al., 2008). Transcriptomic profiling has been used to assess differences between wild type and cultivated sweet potato (Manoharan, 2017). The *MADS* box gene *SRD1* has also been implicated in thickening of storage roots. Noh et al. (2010) found that *SRD1* was involved in the auxin-mediated thickening of storage roots by affecting cell growth in the cambium and metaxlyem. The occurrence of *SRD1* transcripts is mainly in the actively dividing cells, including the vascular and cambium cells, and the increase in endogenous indole-3-acetic acid (IAA) content and three auxin-inducible *AUX/IAA* gene transcripts concomitantly with *SRD1* transcripts suggest the involvement of *SRD1* during the early stage of storage root development. Ravi et al. (2014) described that the genes *Ibkn1* and *Ibkn2* activate cytokinin biosynthesis, which are involved in storage root development. Transcription factors derived from *MADS* box genes *IbMADS1*, *IbMADS3*, *IbMADS4*, and *IbAGL17* induce a signal transduction pathway leading to storage root formation and development.

Wang et al. (2015a) examined the role of gibberellins in carrot root growth and development. They found that gibberellin levels in the roots initially increased and then decreased, but these levels were lower than those in the petioles and leaves. They found that gibberellin level may play a vital role in carrot elongation and expansion, and that carrot growth and development may be influenced by gibberellin biosynthetic genes. Ebener et al. (1993) examined a gene *DcPRP1* and found it was associated with the formation of storage roots in carrot, particularly in response to wounding. They found that *DcPRP1* is linked to secondary root growth and that it can be induced in carrot roots by auxin. Wang et al. (2015b) found 87 hormone-related differentially expressed genes at different stages of carrot root growth. Their findings suggest that hormones may regulate carrot root growth in a phase-dependent manner. Despite these results, much more work must be conducted in carrot and table beet to identify key regulators of storage root elongation; indeed, this is one of the major research gaps for these crops.

Not only in root crops, hormones including auxin, cytokinin, gibberellin, ethylene, and ABA have also been reported to affect fruit shape in diverse crops, the most recent review of which can be found in Wang et al. (2022a). In addition, one of the candidate genes, Solyc08g061930, of the *fs8.1* locus for the elongated and blocky tomato fruit (Grandillo et al., 1996), encodes a protein that regulates cytokinin degradation (Sun et al., 2015), suggesting a function of cytokinin in the tomato fruit shape regulation. Furthermore, recent studies show that *CLASS-II KNOX* (*TKN-II*) genes regulate tomato fruit shape via gibberellin (Shtern et al., 2023), and *CsTRM5* regulates cucumber fruit shape by affecting cell division direction and cell expansion with the involvement of ABA (Xie et al., 2023). In Cucurbits, ethylene regulates transcription factors (*E2F-DP*), *OVATE*, and *TRM5* to determine a round or elongated fruit shape (Boualem et al., 2022). It has been long known that flower sex determination (female *vs.* bisexual flowers) affects fruit shape in Cucurbits (Rosa, 1928; Poole and Grimball, 1945; Kubicki, 1962; Wall, 1967). Generally, fruits from bisexual flowers are rounder than fruit originating from female flowers, although there are some exceptions (Tan et al., 2015). Ethelene is the key hormone in Cucurbit sex determination. Mutations in orthologs of a 1-aminocyclopropane-1-carboxylic acid synthase (ACS) gene is a key regulator of flower sex determination in watermelon (*CitACS4/ClACS7*), cucumber (*CsACS2*), melon (*CmACS7*) and squash (*CpACS27A*) (Boualem et al., 2008; Boualem et al., 2009; Li et al., 2009; Martínez et al., 2014; Boualem et al., 2015; Ji et al., 2015; Boualem et al., 2016; Ji et al., 2016; Manzano et al., 2016; Zhang et al., 2017b). Mutations that lower ACS expression or lead to a reduction in enzyme activity that results in reduced ethylene production promote development of bisexual flowers and thus rounder fruit. In strawberry, differential auxin content and expression of auxin-related genes probably contribute to an elongated fruit mutant (Li et al., 2023). In tomato, a crosstalk between miR319-targeted *TCP4/LANCEOLATE*, and *OVATE* and auxin has been proposed as the factor that establishes fruit shape (Carvalho et al., 2022).

## 4 Developmental aspects of shape regulation and common genetic mechanisms

The shape and size of organs is controlled by coordination of cell growth, cell division, patterning and differentiation (van der Knaap et al., 2014; Sablowski, 2016). The cell wall prevents cellular motility within plant tissues, and thus the direction in which plant cells divide is essential to determine the overall shape. Therefore, plant cells must tightly regulate the frequency and orientation of cell divisions to ensure precise organ growth. Organ shape can be further regulated through directional cell expansion. The timing of gene expression and the spatio-temporal regulation of protein function involved in these processes are essential in growth and morphogenesis, and alterations to expression or function can significantly impact the organ size and shape.

The centrosome in animals organizes the microtubules during different phases of the cell cycle. However, plants don’t have centrosomes and thus microtubules undergo a complex rearrangement during cell division, including the formation of a ring of microtubules encircling the cortex at the onset of mitosis during late G2 called the preprophase band (PPB). The PPB is a cytological landmark of the final division plane and distinguishes the site where the new cell plate will attach at cytokinesis (Müller et al., 2009; Duroc et al., 2010).

In recent years, three plant-specific families of proteins have emerged for their role in regulating fruit, seed, tuber, and leaf morphology. They are the OFPs, including the founding member OVATE in tomato; the OFP-interacting TRMs that are known to associate with a complex that regulate plant cell division; and the SUNs which are members of the IQD family, named after the *sun* locus in tomato (Snouffer et al., 2020). The ubiquity of these protein families indicates a common mechanism that appears to be modular to regulate plant organ shape in many if not all plant organs (Wu et al., 2018).

As mentioned above, perhaps the most convincing evidence to date for universal shape regulation in crops comes from Wu et al. (2018), who showed that OFPs and TRMs are important in controlling fruit, tuber, vegetable, and grain shapes in a variety of crop species including tomato, melon, cucumber, rice, and potato. They suggest that the relative expression and interaction of OFPs and TRMs are critical to the shape of plant organs such as leaves, flowers, fruits, tubers, and roots. They propose that the subcellular localization of these proteins, either as associated with microtubules or in the cytoplasm, can ultimately determine the growth and shape of particular organs. Furthermore, they demonstrate that the relocalization of OFPs and TRMs after their proteins interact implies a regulatory effect of these complexes in the early development of plant organs.

Tomato produces perfect flowers that exhibit five sepals, petals, and stamen each and two to four carpels which fuse during their initiation to form the seed cavities or locules in the ovary. In tomato, carpel primordia emerge from the floral center after the sepal, petal and stamen primordia, approximately 6 days after floral bud initiation. The carpel walls continue to enlarge and elongate while the central portion, comprised of the septum and the central column, results in the formation of the locular cavities (Xiao et al., 2009). Ovule development completes when the flower opens. Ovary shape regulated by OFP-TRM in tomato becomes visible during the earliest stages of carpel development (Kraus, 2019). SUN is highly expressed before and immediately after anthesis and begins impacting shape at this stage. While the exact developmental timing of *OFP*, *TRM*, and *SUN* in fruit shape determination is still under investigation, it is evident that they act early in floral development to regulate cell patterning in the proximo-distal and medio-lateral direction to control ovary shape well before anthesis (Wu et al., 2011; Wu et al., 2018).

Since cell division patterning influences organ shape under the control of OFP-TRM-SUN (Snouffer et al., 2020), it is possible that shape is similarly regulated in other organs, namely at the onset of primordia growth when patterned cell division is most critical. This cellular mechanism of shape explains why the morphology of disparate organs such as tubers and grains are controlled by genes of the same three families. As highlighted above, natural variation of organ shape in diverse crops including rice (*GW7/GL7* (orthologue of *AtTRM1*, Wang et al., 2015c; Wang et al., 2015d), maize (maize *ZmLNG1*, homolog of Arabidopsis *TRMs*; Wang et al., 2022b), potato tuber (*StOFP20* (Wu et al., 2018), cucumber *CsTRM5* (Wu et al., 2018; Wang et al., 2020a, b), *CsSun* (Pan et al., 2017, Wang et al., 2020a, b), pepper CaOvate (Tsaballa et al., 2011), CaOFP20 (Borovsky et al. 2022), CaTRM25 (Taitano, 2020), melon CmOFP13 (Wu et al., 2018, Ma et al., 2022a, Martínez-Martínez et al., 2022), watermelon *ClSUN25-26-27a* (Dou et al., 2018; Legendre et al., 2020), pummelo *CitOFP19* (Wu et al., 2022) and peach *PpOFP2* (Guan et al., 2021).

Studies from genome editing or modification of gene expression of members from these three gene families also support their common function in organ shape regulation. For instance, loss of *SlTRM5* leads to a rounder tomato fruit shape (Wu et al., 2018), which is also true of the knockout of *CsTRM5* in suppressing cucumber fruit elongation (Xie et al., 2023). Knockout of *SlTRM19* causes an elongated tomato fruit shape, and combination of the null mutants *SlTRM19* and *SlTRM17/20a* further elongates the fruit shape (Zhang et al., 2023). Dysfunction of CsOVATE results in a longer cucumber fruit neck (Wang, 2022c). A null mutation in *StOFP20* turns round potato tubers into oval shaped tubers (Ju et al., 2023), and modulating the expression level of this gene alters tuber shape potato (Van Eyck et al., 2022). Ectopic overexpression of peach *PpOFP2* flattens tomato fruit shape (Guan et al., 2021). Overexpression of bottle gourd *LsOVATE1* in tomatoes changes fruit shape (Feng et al., 2023). Overexpression of radish tuberous root shape candidate gene *RsOFP2.3* in Arabidopsis results in shorter but wider hypocotyl and siliques (Wang et al., 2020a). Higher expression of *CitOFP19* leads to pear-shaped ovary and fruit shape in tomato (Wu et al., 2022). Overexpression of watermelon *ClIQD24* in tomato causes an elongated tomato fruit shape (Dou et al., 2022a). Overexpression of melon *IQD*/*SUN* genes (*CmSUN23-24* or *CmSUN25-26-27c*) leads to an elongated fruit shape, while overexpression of *GhIQD10* inhibits cotton fiber length (Ma et al., 2022b; Xu et al., 2023). *CsSUN*-expressed tomato shows elongated fruit shape with increased length and decreased diameter (Li et al., 2022). Paralogs of OFPs have been identified in apple and pear (Li et al., 2019; Ding et al., 2020), although no evidence has yet to associate these genes with shape regulation in these fruit crops. The continuous discovery of the involvement of TRMs, OFPs, and SUNs highlights the ubiquity of this pathway in controlling plant organ shapes.

## 5 Future prospects

Crop wild relatives are considered less relevant for modern breeding efforts with respect to characteristics such as shape, however this view may be unnecessarily limiting. Although they possess obvious utility in providing a point of comparison when studying how shape modifications have proceeded under domestication, crop wild relatives may provide even more value for future shape modifications. Useful allelic variation in wild species has not been fully examined with respect to its potential to support shape modifications. For example, wild populations of *Daucus* and *Beta* species possess supernumerary cambia but have only been selectively subjected to domesticating selection. This suggests that substantial variation may exist in wild populations that have remained untapped (deBock, 1986). Similar scenarios may exist for other root crops such as sweet potato and carrot. Since domestication was not conducted in a systematic way where wild populations were fully sampled for their diversity, wild species populations may contain useful variation for shape modifications that have not yet been tested. Rong et al. (2014) have shown that continued introgression of genes from wild species into domesticated populations may be in part responsible for the modern carrot.

Although traits from crop wild relatives have been mined to improve levels of disease and pest resistance in modern crops, breeders have not typically used these genetic resources for productivity traits (Goldman, 2023a). This is often because wild species chromosome segments may possess unfavorable alleles that can reduce crop productivity. However, this is not necessarily a universal phenomenon: Tanksley and McCouch (1997) crossed wild rice accessions to cultivated inbred lines. They found yield increases in progeny from these crosses, suggesting wild species might be more important contributors to productivity traits than previously thought. Gur and Zamir (2004) reported on traits from wild species of tomato that could increase yield in cultivated tomato even under water deficit conditions. Eshed and Zamir (1995) created an introgression population of *Solanum pennelli* chromosome segments in an elite *Lycopersicon esculentum* background, and found certain introgression lines with high yield and productivity. Johal et al. (2008) developed an approach called MAGIC (mutant-assisted gene identification and characterization) that makes use of mutants or variants in a trait of interest to identify novel variants for the trait. This technique may be able to identify naturally occurring variation that can be incorporated into breeding populations. Naturally occurring variation remains of interest to breeders (Zamir, 2008) not only for disease and pest resistance, but for productivity traits as well.

This research into the potential for yield increases following wide crosses with crop wild relatives suggests that for numerous other quality-associated traits – such as shape – the assumption that wild populations can have only negative impacts is almost certainly not the case. Given the relatively limited sampling of species-wide genetic diversity which has occurred thus far in domestication efforts, it is almost certainly the case that additional diversity at loci associated with the development processes driving shape determination remain unexplored. Furthermore, as concluded by Dohm et al. (2014), the breeding of sugar beet began only in the late 1700’s, with “domesticated” varieties being produced within only a few generations of selection. Redeveloping commercial varieties from wide crosses, particularly when equipped with modern selection and genetic methods, should therefore not be considered an insurmountable task. As such, these populations represent a valuable resource, both as objects of study in their own right, and as material for future crosses aiming at the development of novel shapes and novel genetic backgrounds, to support diversification of existing market classes.

Once orthologs of key shape genes from the gene families OFP, SUN, TRM, and others are identified and markers for these genes are developed, it should be possible to design precision breeding strategies that focus on shape traits. For example, a carrot breeding strategy to modify only the shoulder or tip of the root to a different shape may involve selection in a segregating population based on genotypes of markers known to be associated with particular shoulder or tip shapes. Furthermore, such breeding strategies might not require crosses of very divergent parents. Instead, variants may be identified from within-market class crosses, thereby shortening the time to cultivar development. In addition, identification of marker-shape associations should allow breeders to make inter-market class crosses and precisely select particular shapes while eliminating undesirable segregants. In this way, an important future benefit of defining the genetic control of vegetable crop shape may be both improved breeding efficiency and the use of a wider cross-section of germplasm in breeding efforts.

## Author contributions

IG: Conceptualization, Investigation, Methodology, Project administration, Supervision, Visualization, Writing – original draft, Writing – review & editing. YW: Conceptualization, Data curation, Formal Analysis, Investigation, Methodology, Writing – original draft, Writing – review & editing. AA: Conceptualization, Data curation, Formal Analysis, Investigation, Methodology, Software, Validation, Writing – original draft, Writing – review & editing. SB: Conceptualization, Data curation, Formal Analysis, Investigation, Methodology, Software, Validation, Visualization, Writing – original draft, Writing – review & editing. MO: Conceptualization, Data curation, Formal Analysis, Investigation, Visualization, Writing – original draft, Writing – review & editing. CM: Conceptualization, Data curation, Formal Analysis, Funding acquisition, Investigation, Project administration, Supervision, Writing – review & editing. EvdK: Conceptualization, Data curation, Formal Analysis, Funding acquisition, Investigation, Methodology, Project administration, Software, Supervision, Writing – original draft, Writing – review & editing.
